# The Situation and the Medical Profession

**Published:** 1903-12

**Authors:** F. Paschal


### The Situation and the Medical Profession.

BY DR. F. PASCHAL.

*Editor Texas Medical Journal:*

The organization holding together the medical profession of San Antonio has, during the past five or six eventful weeks, been put to a severe test, and I am glad to be able to report that the plan upon which that organization was accomplished has not been found wanting.

As soon as Drs. Tabor and Murray had demonstrated beyond a doubt that yellow fever existed in San Antonio and had called upon the profession for support in an effort to control the disease, the strongest possible influences were exerted by politicians, with selfish ends to gain, and by certain other misguided citizens, to break up the profession into factions for the purpose of destroying its influence. No amount of bluffing or cajoling, however, could influence the physicians of the city to desert the path of honor and duty for the purpose of gratifying personal grudges and promoting selfish ends. Almost every man showed a willingness to put aside personal likes and dislikes and to stand with the majority for the good of the city and the honor of the profession. In scarcely a single instance did the desire to discredit and humiliate a rival influence a physician to turn his back upon what he knew to be his plain duty. I do not pretend to say that the four or five physicians who did oppose Dr. Tabor and the united profession of the city, were dishonest in doing so. They were certainly entitled to their own opinion as to the right and wrong course under the circumstances.

The smallness of their number, however, illustrated how few were misled into following the leadership of men who, for sake of personal aggrandizement, refused to co-operate with the duly constituted authorities in their efforts to eradicate a disease which had at least been officially declared to be yellow fever.

The following is a list of those who actively co-operated with the authorities:

PHYSICIANS WHO ACTIVELY CO-OPERATED WITH THE HEALTH AUTHORITIES.

Drs. F. Herff, A. Herff, J. Herff, J. Braunnagle, F. H. Hicks, M. J. Bleim, T. T. Jackson, J. S. Lankford, L. L. Shropshire, Robt. E. Moss, B. F. Kingsley, G. G. Watts, W. B. Russ, T. P. Lloyd, P. Baldesarelli, W. L. Barker, H. D. Barnitz, J. H. Bell, E. Bennett, L. M. Berg, J. H. Bindley, H. A. Blair, S. Burg, A. Burke, J. H. Burleson, R. Caffery, H. J. Chapman, E. C. Clavin, P. J. Cleary, E. Cross, P. J. A. Cleary, J. L. Davis, C. M. Decke, A. D. Dupuy, E. H. Elmendorf, E. C. Evans, R. A. Goeth, M. L. Graves, J. H. Harrison, E. F. Hertsberg, J. M. Hooper, E. T. Hughes, C. E. Keller, J. W. Kenney, J. Kingsley, S. T. Lowry, W. E. Luter, C. F. Mason, F. Paschal, A. S. McDaniel, G. H. Moody, J. H. Moore, T. E. Moore, J. P. Oldham, A. O'Malley, R. L. Porter, C. W. Rabb, R. Robinson, J. P. Rote, F. Terrell, S. Trevino, H. J. Trollinger, C. Warfield, J. Watts, R. L. Withers, W. M. Wolf, F. E. Young, J. Greenwood, W. L. Allinson, Geo. Fegan, C. C. Quillian, Chas. W. Taylor, G. G. Clifford, J. E. Gardiner, J. D. Bell, J. F. Hines, R. Menger and C. A. Wilson.

The physicians who did not co-operate with the health authorities are five in number, and their names were placed on "the roll of honor."

